# Quantitative background parenchymal enhancement to predict recurrence after neoadjuvant chemotherapy for breast cancer

**DOI:** 10.1038/s41598-019-55820-5

**Published:** 2019-12-16

**Authors:** Sebastien Moliere, Isabelle Oddou, Vincent Noblet, Francis Veillon, Carole Mathelin

**Affiliations:** 1 Department of Women’s imaging, Strasbourg University Hospital, Strasbourg, France; 2 ICube - IMAGeS, UMR 7357 Illkirch, France; 3 Fédération de Médecine Translationnelle de Strasbourg (FMTS), Strasbourg, France; 4 Department of Radiology, Strasbourg University Hospital, Strasbourg, France; 5 Department of Gynecology and Obstetrics, Strasbourg University Hospital, Strasbourg, France; 6 University of Strasbourg, IGBMC, CNRS UMR 7104, Inserm, U964 Illkirch-Graffenstaden, France

**Keywords:** Prognostic markers, Breast cancer

## Abstract

Breast background parenchymal enhancement (BPE) is an increasingly studied MRI parameter that reflects the microvasculature of normal breast tissue, which has been shown to change during neoadjuvant chemotherapy (NAC) for breast cancer. We aimed at evaluating the BPE in patients undergoing NAC and its prognostic value to predict recurrence. MRI BPE was visually and quantitatively evaluated before and after NAC in a retrospective cohort of 102 women with unilateral biopsy-proven invasive breast cancer. Pre-therapeutic BPE was not predictive of pathological response or recurrence. Quantitative post-therapeutic BPE was significantly decreased compared to pre-therapeutic value. Post-therapeutic quantitative BPE significantly predicted recurrence (HR = 6.38 (0.71, 12.06), p < 0.05).

## Introduction

Neoadjuvant chemotherapy (NAC) is increasingly used in locally advanced breast cancers and in women at high risk of occult distant metastases^1^.

Tumor response after NAC is assessed pathologically^2^, but imaging biomarkers based on breast dynamic-contrast enhanced MRI (DCE-MRI), such as the size of residual enhancing tumor after NAC, have shown very promising leads for non-invasive monitoring of NAC efficacy and prediction of long-term prognosis^3–5^.

Background parenchymal enhancement – the enhancement of non-tumor breast tissue – is a promising MRI biomarker that is linked to overall breast cancer risk^6,7^. Its variation during NAC has been related to pathological response or patient outcome^8,9^.

However, the biological determinants of BPE are poorly known, except for its hormonal dependence, that accounts for the high variability of BPE in premenopausal women^10^.

We hypothesized that post-chemotherapy BPE would evaluate residual biological activity of the breast and help define a subgroup of patients at high risk for recurrence.

In this study, we studied the ability of post-chemotherapy BPE to predict the risk of recurrence. We used quantitative evaluation of BPE to determine if subtle changes in BPE is associated with different prognosis.

## Material and Method

### Population

All consecutive women with a biopsy-proven breast cancer, diagnosed between 01.01.2012 and 01.01.2017, and treated with NC plus surgery in our institution, with available pre- and post-chemotherapy MRI, were retrospectively included.

3 patients were excluded from the analysis because of missing radiology or pathology data.

On the 102 remaining patients, 84 received a combination of Epirubicine, 5-Fluoro-uracile, Cyclophosphamide, 51 received additional weekly treatment with taxane and 33 patients received Trastuzumab therapy.

Pre-operative recorded data included: patient’s age, clinical TNM stage at diagnosis, tumor pathology and molecular subtype^11^.

All surgical breast specimens were analyzed in the pathology department of our institution. A pathological complete response (pCR) was defined as the absence of residual invasive tumor in both the breast and the axillary nodes.

Each patient underwent post-operative annual follow-up including clinical examination, mammography, thoraco-abdominal imaging and determination of serum CA 15-3 value.

Recurrence was characterized as local (recurrence in the original tumor bed with the same histopathologic features of the primary tumor), regional (recurrence in the ipsilateral axillary, internal mammary or supraclavicular or infraclavicular nodes) or distant (metastasis in all other locations), and the length of recurrence-free survival (RFS) was defined as the time from surgery to recurrence or to the last follow-up in patients without evidence of recurrence.

### MRI acquisition

All included patients underwent dynamic contrast-enhanced (DCE) MRI before treatment and after the last cycle of chemotherapy, with a mean time to surgery of 15 days (2–35).

MRI were performed on a 1.5 T unit with a dedicated breast coil (Aera, Siemens Healthcare). The standard protocol included an unenhanced axial 3D fat-suppressed T1-weighted sequence and five consecutive post-contrast series (0.1 mmol/kg of gadoteric acid, Dotarem, Guerbet, Roissy, France). The first post-contrast acquisition was centered at 45 seconds after contrast material injection, with a temporal resolution of 90 seconds, the late phase was centered at 6 minutes. Scanning parameters were: acquisition time 90 s, repetition time (TR)/echo time (TE): 4.41/1.78, flip angle 10°, field of view (FOV) 359, slice thickness 1.5 mm.

### MRI visual analysis

Breast MRI were independently analyzed by two readers (SM: reader 1, IO: reader 2 with respectively 6 and 3 years of breast MRI experience). Readers were blinded to pathology data.

A radiological complete response was defined as the absence of residual tumor enhancement at the early phase. Disagreements between readers were resolved by consensus.

Both readers independently assessed MRI breast density (fibroglandular tissue, FGT) by using the four BI-RADS categories (A, B, C and D) and BPE by using the four BI-RADS categories (1 or minimal, 2 or mild, 3 or moderate, 4 or marked) on subtracted enhanced series^12^. A second visual evaluation of FGT and BPE was also done by reader 1 after a 6-month wash-out period. Disagreements between readers were resolved by consensus.

Inter-reader and intra-reader variability of FGT and BPE visual assessment were evaluated.

### MRI quantitative analysis

**Figure 1:**
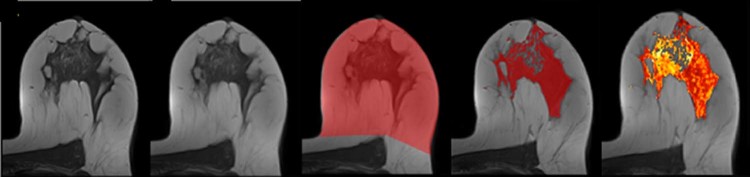
Steps of quantitative assessment of BPE. 1: native image, 2: deformable registration of the postcontrast time series to the precontrast time series, 3: supervised delimitation of chest wall, 4: segmentation of FGT by thresholding, 5: parametric map of BPE20%.

For quantitative assessment of FGT we used a semi-automated computer segmentation of the non-tumor breast on T1-weighted pre-contrast images (Fig. 1), including: i correction of field inhomogeneities using N4 method^13^, ii deformable registration of the postcontrast time series to the precontrast time series, using ANTS software^14^, iii supervised delimitation of chest wall, in consensus by two operators, and iv. segmentation of FGT by thresholding based on fuzzy C-means.

We then defined quantitative FGT as the proportion of FGT volume (V_FGT_) to the whole breast volume (V_Breast_):

$$FGT=\frac{{V}_{FGT}}{{V}_{Breast}}x\,100$$

For BPE quantitative assessment, we used a previously published method [15].

For each voxel, we evaluated R_voxel_, the ratio between the voxel’s intensity in the substracted image of the early phase (I_post_ − I_pre_) and its intensity in the pre-contrast image (I_pre_):

$${R}_{voxel}=\frac{{I}_{post}-{I}_{pre}}{{I}_{pre}}$$

We then calculated V_BPE_, the total volume of the enhancing voxels over the FGT region that had a relative difference equal to or greater than a predefined enhancement ratio threshold, that we set at 20% in accordance with previous studies^15,16^:

$${V}_{BPE}=\sum _{voxel\,\in \,FGT\,}{V}_{voxel}|{R}_{voxel}\ge 20 \% $$

BPE20% was then defined as the proportion of this volume to the whole FGT volume.

$$BPE20 \% =\frac{{V}_{BPE}}{{V}_{FGT}}\times 100$$

Intrasubject change of BPE20% was defined as:

$${\Delta }BPE20 \% =\,\frac{BPE20{ \% }_{post}-BPE20{ \% }_{pre}}{BPE20{ \% }_{pre}}\times 100$$

where BPE20%_pre_ is the value of BPE on the pretherapeutic MRI, and BPE20%_post_ the value of BPE on the post-therapeutic MRI.

### Statistics

Comparisons were done using Student t test. Inter- and intra-rater reliability were assessed with Fleiss’ kappa method. Correlation between visual and quantitative FGT and BPE was analyzed with Pearson’s method.

Multivariate Cox regression was used for disease-free survival (DFS) analysis. Patients lost to follow-up were excluded from the analysis. All Cox proportional hazards results were reported as estimated hazard ratios, 95% confidence intervals and likelihood ratio test p-values. Statistical analyses were performed using Python’s module Statsmodels (https://www.statsmodels.org) and Lifelines (http://lifelines.readthedocs.io/) for DFS analysis. A statistical significance level of p = 0.05 was used throughout.

### Ethics

This study received Institutional Review Board approval (Comité de Protection des Personnes Est). Informed consent was obtained for each participant and the study followed the relevant guidelines: Strengthening the Reporting of Observational Studies in Epidemiology (STROBE).

## Results

### Characteristics of the population

Most of the patients had intermediate- or high-grade tumors (96%), histological subtypes were either luminal (41%), HER2-enriched (31%) or basal (28%).

Most of the patients were either post-menopausal (47%) or had chemotherapy-induced amenorrhea (30%).

Pathological complete response was obtained in 27 patients.

Table 1 describes the characteristics of the population.

**Table 1:** Clinical and Pathological Characteristics.

	Total population (N = 102)
Mean age at diagnosis	49,8
Pre-menopausal at diagnosis	54
Chemotherapy-induced amenorrhea	31
**Breast tumor stage**	
cT1	8
cT2	59
cT3	25
cT4	10
Pre-therapeutic lymph node involvement	42
**SBR grade I/II/III**	
Grade I	4
Grade II	34
Grade III	64
**Immunohistochemical subgroups**	
Luminal A	9
Luminal B	33
ERB-B2 overexpression	32
Basal type	28
Pathological complete response	27

Abbreviations: SBR Scarff Bloom Richardson, ERBB2 erythroblastic oncogene B 2.

°Definition of hormone-receptor positivity was H-score (percentage of positive cells x intensity of immunoreactivity) superior or equal to 10.

°°Definition of HER2-receptor positivity was 3 + IHC score (strong complete membrane staining in > 10% of tumor cells) or 2 + IHC score with amplification on ISH.

### Inter and intra reader correlation of visual parameters

There was excellent agreement between readers for visual evaluation of FGT (kappa = 0.85, p < 0.001), but only fair agreement for visual evaluation of BPE (kappa = 0.44, p < 0.001 for pre-therapeutic imaging and kappa = 0.41, p < 0.001 for post-therapeutic imaging). Visual BPE had fair to good intra-rater reliability (kappa = 0.64, p < 0.001 for pre-therapeutic imaging, kappa = 0.50, p < 0.001 for post-therapeutic imaging).

### Correlation between visual and quantitative parameters

Pre and post-therapeutic quantitative FGT were almost perfectly correlated (r = 0.98 p < 0.001).

Visual and quantitative evaluation of pretherapeutic FGT were strongly correlated (r = 0.78 p < 0.001).

Quantitative BPE was reduced on average by 37% after NAC. The decrease was higher in premenopausal women (48%).

No difference was found in pretherapeutic BPE, post-therapeutic BPE or intrasubject change in BPE across different tumor subtypes.

BPE decrease after chemotherapy was correlated with post-therapeutic quantitative BPE value (r = −0.57, p < 0.001).

Visual and quantitative evaluation of BPE were fairly correlated in the pre-therapeutic imaging (r = 0.51 p < 0.001) and weakly correlated in the post-therapeutic imaging (r = 0.27 p = 0.008).

### Pathological response after NAC

Comparison between pCR and non-pCR groups is shown in Table 2.

**Table 2:** Univariate analysis.

	pCR (N = 27) n (%)	Non pCR (N = 75) n (%)	p value
**Clinical data**			
Mean age at diagnosis	49.41 ± 10.5	49.89 ± 10.1	ns
Mean Body Mass Index	26.36 ± 4.7	27.85 ± 6	ns
Post-menopausal	13 (48.1)	34 (45.3)	ns
Pre-therapeutic lymph node involvement	17 (63.0)	53 (70.6)	0.07
Mastitis	0	8 (10.7)	0.03
**Pre-therapeutic biopsy**			
SBR grade			<0.01
*grade I*	1 (3.7)	3 (4)	
*grade II*	2 (7.4)	32 (42.7)	
*grade III*	24 (88.9)	40 (53.3)	
Immunohistochemical subgroups			<0.01
*Luminal A*	1 (11.1)	8 (10.7)	
*Luminal B*	5 (18.5)	28 (37.3)	
*Erb-B2 overexpression*	8 (29.6)	24 (32)	
*Basal type*	13 (48.1)	15 (20)	
Mean Ki67	56.89 (24.7)	40.23 (22.8)	0.03
**Pre-therapeutic MRI**			
Largest tumor diameter (mm)	28.31 ± 12	37.51 ± 18,7	<0.01
Tumor necrosis	10 (37)	25 (33.3)	ns
Peritumoral edema	16 (22.2)	33 (44)	ns
Visual Fibroglandular Tissue*			ns
*BIRADS A*	7 (25.9)	23 (30.6)	
*BIRADS B*	11 (40.7)	31 (41.3)	
*BIRADS C*	4 (14.8)	12 (16)	
*BIRADS D*	5 (18.5)	7 (9.3)	
Quantitative Fibroglandular Tissue	10 ± 9	10 ± 9	ns
Visual BPE*			ns
1	10 (37)	25 (33.3)	
2	8 (29.6)	21 (28)	
3	4 (14.8)	13 (17.3)	
4	5 (18.5)	16 (21.3)	
Mean quantitative BPE	8.1 ± 1.8	8.2 ± 1.7	ns
Post-therapeutic MRI			
Radiological complete response	25 (92.6)	12 (16)	<0.01
Visual BPE*			ns
1	21 (77.8)	56 (74.7)	
2	5 (18.5)	7 (9.3)	
3	1 (3.7)	8 (10.7)	
4	0	4 (5.3)	
Mean quantitative BPE	5.4 ± 2.7	4.8 ± 2.0	ns

ns: non-significant.

*as evaluated by reader 1.

SBR Scarff-Bloom-Richardson.

ERBB2 erythroblastic oncogene B 2.

BPE Breast parenchymal enhancement.

Radiological complete response had a 93% (CI95% 83–100) sensitivity and 84% (CI95% 76–92) specificity for predicting pCR.

There was no significant difference between complete responders and non-complete responders in term of pre and post-therapeutic BPE.

### Disease-free survival

Median follow-up was 37 months (15–59). One patient was lost to follow-up. During follow-up there were 2 local recurrences, 3 regional recurrences and 10 distant metastases. Recurrences were found in 1 luminal A tumor, 7 luminal B tumors, 2 HER2 tumors and 5 basal type tumors (Table 3).

**Table 3:** Recurrences during follow-up period.

Patient	Age	Menopausal status	Histological type, grade and molecular subtype	cTNM	NAC regimen	Pathological complete response	Type of recurrence
1	50	post-menopausal	ductal invasive carcinoma, grade 3, Erb-B2 overexpression	cT3N0	taxane, trastuzumab	no	regional
2	48	pre-menopausal	ductal invasive carcinoma, grade 3, basal type	cT3N2	taxane, epirubicine,	no	distant
3	55	post-menopausal	ductal invasive carcinoma, grade 3, basal type	cT1N0	taxane, epirubicine,	yes	distant
4	53	post-menopausal	ductal invasive carcinoma, grade 2, luminal B type	cT4DN0	taxane, epirubicine, trastuzumab	no	regional
5	43	pre-menopausal	lobular invasive carcinoma, grade 2, luminal B type	cT2N0	taxane, epirubicine,	no	distant
6	44	pre-menopausal	ductal invasive carcinoma, grade 3, luminal B type	cT4DN1	taxane, epirubicine, trastuzumab	no	local
7	50	post-menopausal	ductal invasive carcinoma, grade 3, basal type	cT2N1	taxane, epirubicine,	yes	distant
8	30	pre-menopausal	ductal invasive carcinoma, grade 2, Erb-B2 overexpression	cT4DN2	taxane, trastuzumab	no	distant
9	48	pre-menopausal	ductal invasive carcinoma, grade 3, basal type	cT2N1	taxane, epirubicine,	no	distant
10	55	post-menopausal	ductal invasive carcinoma, grade 3, basal type	cT2N0	taxane, epirubicine,	no	distant
11	55	pre-menopausal	ductal invasive carcinoma, grade 2, luminal A type	cT4BN0	taxane, epirubicine,	no	distant
12	54	post-menopausal	ductal invasive carcinoma, grade 3, luminal B type	cT2N1	taxane, epirubicine,	no	distant
13	53	pre-menopausal	ductal invasive carcinoma, grade 2, luminal B type	cT4BN1	taxane, epirubicine,	no	regional
14	37	pre-menopausal	lobular invasive carcinoma, grade 2, luminal B type	cT2N0	taxane, epirubicine, trastuzumab	yes	distant
15	63	post-menopausal	ductal invasive carcinoma, grade 3, luminal B type	cT1N1	taxane, epirubicine, trastuzumab	yes	local

**Figure 2:**
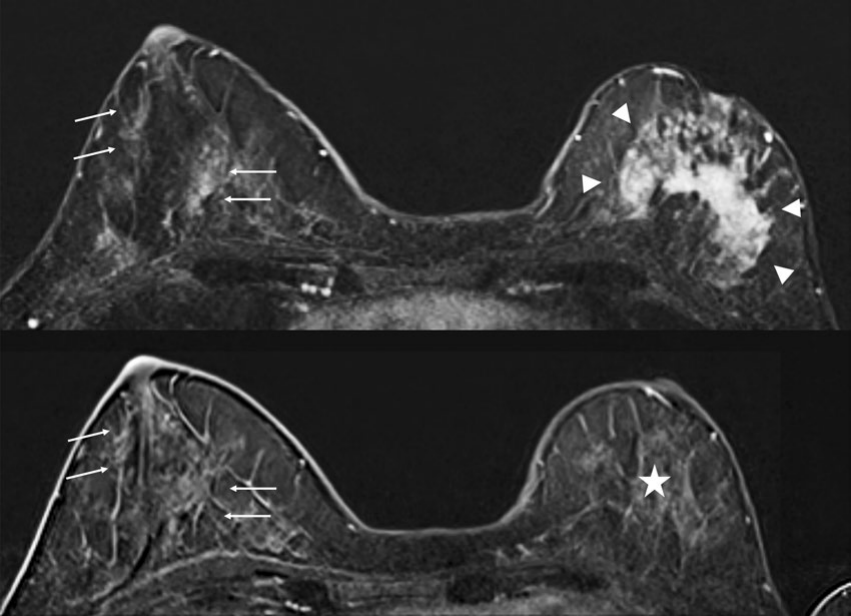
Breast MRI of a 45-year-old premenopausal woman with grade II luminal B HER 2 positive cT2N2 ductal invasive carcinoma relapsing with bone metastases 2 years after surgery, despite having achieved pathological complete response with neoadjuvant chemotherapy. Pretherapeutic MRI (A) showed a 5 cm tumor in the left breast (arrowheads) and moderate BPE, as evaluated in the right breast (arrows). Post-therapeutic MRI (B) showed no residual enhancing tumor in the left breast (star) with persisting BPE (arrows).

Multivariate Cox analysis (Table 4) showed a strong positive association between the quantitative post-therapeutic BPE and the risk of recurrence (HR = 6.38 (0.71, 12.06) p < 0.05). An illustration of these findings by a case is shown in Fig. 2.

**Table 4:** Multivariate Cox regression analysis for recurrence (number of events = 15).

	Hazard Ratio (95% confidence interval)	p value
**Clinical data**		
Age at diagnosis	0.042 (−0.022, 0.11)	0.19
Pre-therapeutic lymph node involvement	0.0411 (−1.74, 1.82)	0.96
Mastitis	3.8822 (0.84, 6.93)	**0.012**
**Breast biopsy**		
Luminal type	Reference	
HER2-positive	−2.46 (−4.65, 0.27)	**0.027**
Basal type	−0.73 (−2.50, 1.03)	0.41
**Pre-therapeutic MRI features**		
Largest tumor diameter (mm)	0.027 (−0.014, 0.067)	0.19
Quantitative BPE	0.038 (−0.044, 0.12)	0.19
**Post-therapeutic MRI features**		
Largest residual tumor diameter (per mm)	0.055 (0.0067, 0.10)	**0.025**
Quantitative BPE	6.38 (0.71, 12.06)	**0.027**
Pathological complete response	−1.55 (−3.53, 0.42)	0.12

**Figure 3:**
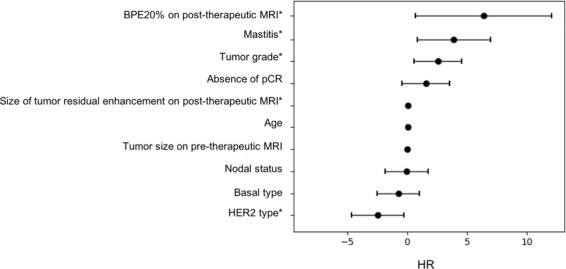
Hazard ratio (HR) of breast cancer recurrence after NAC. Statistically significant results are shown with a star (*).

Other parameters significantly linked to local or distant recurrence risk were: mastitis at presentation, high tumor grade, HER2 negativity, larger residual enhancing tumor diameter on post-therapeutic MRI (Fig. 3).

## Discussion

Post-therapeutic quantitative BPE is an independent predictor of recurrence after NAC.

This results broaden other published results, that have shown BPE to be linked with breast cancer risk^16,15^, breast metabolic activity^17^, as well as therapeutic response^8,18–20^.

The precise physiology of BPE is poorly known. While independent of breast density, it has been shown to be highly dependent on the breast fibroglandular tissue’s exposure to hormones^21^. Chemotherapy frequently causes transient or permanent ovarian failure, which may have *per se* a therapeutic effect on hormone-positive breast cancer [22]^22^.

In our study, amenorrhea was noted in 77% of the premenopausal women undergoing post-NAC MRI, but the degree of ovarian suppression probably differs between patients. BPE could thus give quantitative information about chemotherapy-induced ovarian suppression in premenopausal women^16^, as well as residual baseline estrogen levels in post-menopausal women^23^.

Ultimately, the intensity of BPE ultimately reflects the density and permeability of blood and lymphatic vessels in the breast, which are known to be important cofactors of breast tumor growth, angiogenesis, metastasis and immune response^24–26^. Along with their cytotoxic effect on tumor cells, chemotherapy drugs have a direct effect on endothelial cells^18^, thus decreasing BPE. This decrease may be an indirect marker of chemotherapy action.

Quantitative assessment of BPE allows an objective appreciation of the microvasculature of the breast gland and is able to highlight subtle changes of vessels density and permeability, not easily detected by visual evaluation^27^, especially in the post-chemotherapy setting.

In fact, there was only moderate correlation between quantitative and visual evaluation. This is certainly explained by the significant inter-observer variability of the 4-grade visual assessment, the presence of motion-induced artifacts (as the visual evaluation was done on subtracted uncorrected series)^28^ as well as the difficulty to visually assess BPE in mainly fatty breasts.

Tumor immunohistochemical subtype was not significantly linked to pretherapeutic or post-therapeutic quantitative BPE, nor BPE change under chemotherapy.

On the contrary to a previous study^18^, no influence of tumor subtype on the prognostic value of BPE could be showed in this study, because of the small number of events in each subtype (1 for luminal A tumor, 7 for luminal B, 2 for HER2 and 5 for basal).

While our results tend to demonstrate the potentially general prognostic value of quantitative BPE evaluation, larger studies are needed to determine in which oncological situations quantitative BPE could be an optimal biomarker.

Interestingly, we did not find any significant change in the breast density after NAC. While a decrease of breast density after chemoprevention has been reported in many publications, change in breast density after chemotherapy has rarely been studied^29,30^, only one study described a decrease in breast density (in average a 10–12% decrease) following neoadjuvant chemotherapy^29^. Some explanations can be raised. First, we did not exclude post-menopausal women and women with extremely fatty breast from the analysis, while hese women – roughly half of the study population - are highly unlikely to have a significant hormonal-related decrease in breast density. Furthermore, the change in breast density implies extensive histological changes, with fatty involution of the glandular tissue, which may take more time than the usual 4 months between pretherapeutic and post-therapeutic MRI, on the contrary to BPE which has proved to be changing on a much faster pace, during the menstruation cycle^21^.

We found that quantitative BPE was correlated to disease-free survival, independently of pCR. Pathological complete response did not reach statistical significance, probably due to a small number of events during the follow-up period.

Chen *et al*.^18^ showed a correlation between pathological response and early decreased of BPE after one cycle of NAC in an ER-negative subgroup. In fact, we chose to evaluate BPE at the end of the treatment, as routinely done in our institution, which may not bring the same information.

In the setting of neoadjuvant chemotherapy, the prognostic value of an imaging biomarker, independently of pathological complete response, has already been published, for MRI-assessed post-therapeutic functional tumor volume^5^.

Our study highlights the role of quantitative MRI assessment of normal breast tissue’s microvascularization after chemotherapy for predicting prognosis, independently of pathological analysis of the tumor. If validated by larger studies, this biomarker could in particular prove useful to tailor the type and the rate of post-operative follow-up.

Our study has limitations, among them the retrospective nature of this study, the relatively small population size and the limited number of events during the follow-up period. The inhomogeneity of treatment, with some patients having adjuvant hormone therapy during the follow-up period, may also limit the extrapolation of the results and should prompt further larger prospective studies.

## Conclusion

Quantitative BPE is strongly linked to recurrence after NAC. More biological and clinical studies are needed to compare the different measurement methods with their underlying pathophysiological mechanisms.

## References

[1] Kaufmann M (2012). Recommendations from an international consensus conference on the current status and future of neoadjuvant systemic therapy in primary breast cancer. Ann. Surg. Oncol..

[2] Cortazar P (2014). Pathological complete response and long-term clinical benefit in breast cancer: the CTNeoBC pooled analysis. Lancet Lond. Engl..

[3] Abramson RG (2015). Methods and Challenges in Quantitative Imaging Biomarker Development. Acad. Radiol..

[4] Lobbes MBI (2013). The role of magnetic resonance imaging in assessing residual disease and pathologic complete response in breast cancer patients receiving neoadjuvant chemotherapy: a systematic review. Insights Imaging.

[5] Hylton NM (2016). Neoadjuvant Chemotherapy for Breast Cancer: Functional Tumor Volume by MR Imaging Predicts Recurrence-free Survival-Results from the ACRIN 6657/CALGB 150007 I-SPY 1 TRIAL. Radiology.

[6] King V (2011). Background parenchymal enhancement at breast MR imaging and breast cancer risk. Radiology.

[7] Dontchos BN (2015). Are Qualitative Assessments of Background Parenchymal Enhancement, Amount of Fibroglandular Tissue on MR Images, and Mammographic Density Associated with Breast Cancer Risk?. Radiology.

[8] Hattangadi J (2008). Breast Stromal Enhancement on MRI Is Associated with Response to Neoadjuvant Chemotherapy. Am. J. Roentgenol..

[9] Preibsch H (2016). Background parenchymal enhancement in breast MRI before and after neoadjuvant chemotherapy: correlation with tumour response. Eur. Radiol..

[10] Pujara Akshat C., Mikheev Artem, Rusinek Henry, Gao Yiming, Chhor Chloe, Pysarenko Kristine, Rallapalli Harikrishna, Walczyk Jerzy, Moccaldi Melanie, Babb James S., Melsaether Amy N. (2017). Comparison between qualitative and quantitative assessment of background parenchymal enhancement on breast MRI. Journal of Magnetic Resonance Imaging.

[11] Perou CM (2000). Molecular portraits of human breast tumours. Nature.

[12] Morris, E., Comstock, C. & Lee, C. ACR BI-RADS Magnetic Resonance Imaging. in *ACR BI-RADS® Atlas*, *Breast Imaging Reporting and Data System* (2013).

[13] Tustison NJ (2010). N4ITK: improved N3 bias correction. IEEE Trans. Med. Imaging.

[14] Avants BB, Epstein CL, Grossman M, Gee JC (2008). Symmetric diffeomorphic image registration with cross-correlation: evaluating automated labeling of elderly and neurodegenerative brain. Med. Image Anal..

[15] Wu S (2017). DCE-MRI Background Parenchymal Enhancement Quantified from an Early versus Delayed Post-contrast Sequence: Association with Breast Cancer Presence. Sci. Rep..

[16] Wu S (2015). Quantitative assessment of background parenchymal enhancement in breast MRI predicts response to risk-reducing salpingo-oophorectomy: preliminary evaluation in a cohort of BRCA1/2 mutation carriers. Breast Cancer Res..

[17] Mema E (2018). Does breast MRI background parenchymal enhancement indicate metabolic activity? Qualitative and 3D quantitative computer imaging analysis. J. Magn. Reson. Imaging JMRI.

[18] Chen JH (2015). Background Parenchymal Enhancement of the Contralateral Normal Breast: Association with Tumor Response in Breast Cancer Patients Receiving Neoadjuvant Chemotherapy. Transl. Oncol..

[19] van der Velden BHM, Dmitriev I, Loo CE, Pijnappel RM, Gilhuijs KGA (2015). Association between Parenchymal Enhancement of the Contralateral Breast in Dynamic Contrast-enhanced MR Imaging and Outcome of Patients with Unilateral Invasive Breast Cancer. Radiology.

[20] Olshen A (2018). Features of MRI stromal enhancement with neoadjuvant chemotherapy: a subgroup analysis of the ACRIN 6657/I-SPY TRIAL. J. Med. Imaging Bellingham Wash.

[21] Kuhl CK (1997). Healthy premenopausal breast parenchyma in dynamic contrast-enhanced MR imaging of the breast: normal contrast medium enhancement and cyclical-phase dependency. Radiology.

[22] Zhou Q, Yin W, Du Y, Shen Z, Lu J (2015). Prognostic impact of chemotherapy-induced amenorrhea on premenopausal breast cancer: a meta-analysis of the literature. Menopause N. Y. N.

[23] Brooks Jennifer D., Sung Janice S., Pike Malcolm C., Orlow Irene, Stanczyk Frank Z., Bernstein Jonine L., Morris Elizabeth A. (2018). MRI background parenchymal enhancement, breast density and serum hormones in postmenopausal women. International Journal of Cancer.

[24] Artacho-Cordón A, Artacho-Cordón F, Ríos-Arrabal S, Calvente I, Núñez MI (2012). Tumor microenvironment and breast cancer progression. Cancer Biol. Ther..

[25] Kang DK, Kim EJ, Kim HS, Sun JS, Jung YS (2008). Correlation of whole-breast vascularity with ipsilateral breast cancers using contrast-enhanced MDCT. AJR Am. J. Roentgenol..

[26] Karaman S, Detmar M (2014). Mechanisms of lymphatic metastasis. J. Clin. Invest..

[27] Kim JY (2015). Enhancement parameters on dynamic contrast enhanced breast MRI: do they correlate with prognostic factors and subtypes of breast cancers?. Magn. Reson. Imaging.

[28] You C (2018). The Assessment of Background Parenchymal Enhancement (BPE) in a High-Risk Population: What Causes BPE?. Transl. Oncol..

[29] Chen J-H (2010). Decrease in breast density in the contralateral normal breast of patients receiving neoadjuvant chemotherapy: MR imaging evaluation. Radiology.

[30] Toledano A (2006). Concurrent administration of adjuvant chemotherapy and radiotherapy after breast-conserving surgery enhances late toxicities: long-term results of the ARCOSEIN multicenter randomized study. Int. J. Radiat. Oncol. Biol. Phys..

